# Cytotoxic Evaluation and Anti-Angiogenic Effects of Two Furano-Sesquiterpenoids from *Commiphora myrrh* Resin

**DOI:** 10.3390/molecules25061318

**Published:** 2020-03-13

**Authors:** Ali S. Alqahtani, Fahd A. Nasr, Omar M. Noman, Muhammad Farooq, Tariq Alhawassi, Wajhul Qamar, Ali El-Gamal

**Affiliations:** 1 Department of Pharmacognosy, College of Pharmacy, King Saud University, Riyadh 11451, Saudi Arabia; aelgamel@ksu.edu.sa; 2 Medicinal, Aromatic and Poisonous Plants Research Center, College of Pharmacy, King Saud University, Riyadh 11451, Saudi Arabia; onoman@ksu.edu.sa; 3 Medication Safety Research Chair, College of Pharmacy, King Saud University, Riyadh 11451, Saudi Arabia; tarriq@ksu.edu.sa; 4 Bioproducts Research Chair, College of Science, Department of Zoology, King Saud University, Riyadh 11451, Saudi Arabia; fmuhammad@ksu.edu.sa; 5 Department of Clinical Pharmacy, College of Pharmacy, King Saud University, Riyadh 11451, Saudi Arabia; 6 Central Laboratory, College of Pharmacy, King Saud University, Riyadh 11451, Saudi Arabia; wqidris@ksu.edu.sa

**Keywords:** *commiphora myrrh*, furano-sesquiterpenoids, apoptosis, cell cycle, angiogenesis, zebrafish

## Abstract

*Commiphora myrrh* resin (Myrrh) has been used in traditional Arabic medicine to treat various inflammatory diseases. Two furano-sesquiterpenoids, 2-methoxyfuranodiene (CM1) and 2-acetoxyfuranodiene (CM2), were isolated from the chloroform fraction of the ethanolic extract of Arabic *Commiphora myrrh* resin. The cytotoxicity of the compounds was evaluated using human liver carcinoma, breast cancer cells (HepG2 and MCF-7, respectively) and normal human umbilical vein endothelial cells (HUVECs) cell lines. The development toxicity and anti-angiogenic activity of both compounds were also evaluated using zebrafish embryos. Cell survival assays demonstrated that both compounds were highly cytotoxic in HepG2 and MCF7 cells, with IC_50_ values of 3.6 and 4.4 µM, respectively. Both compounds induced apoptosis and caused cell cycle arrest in treated HepG2 cells, which was observed using flow cytometric analysis. The development toxicity in zebrafish embryos showed the chronic toxicity of both compounds. The toxicity was only seen when the embryos remained exposed to the compounds for more than three days. The compound CM2 showed a significant level of anti-angiogenic activity in transgenic zebrafish embryos at sublethal doses. Thus, we demonstrated the cytotoxic properties of both compounds, suggesting that the molecular mechanism of these compounds should be further assessed.

## 1. Introduction

Cancer is the second leading cause of death worldwide. In 2018, 18.1 million new cases and 9.6 million deaths were reported globally [1]. Medicinal plants, including plant resins, are known to contain compounds that may serve as new cytotoxic compounds to combat cancer [2]. *Commiphora myrrh* (family: Burseraceae) is a medicinal plant, which is known to produce a type of aromatic oleo-gum resin known as myrrh, that grows in Yemen and the southern regions of Saudi Arabia [3]. Over the years, it has been claimed that myrrh is very safe for human and animal use, and the medicinal properties of the biologically active constituents have been described in various in vitro and in vivo studies [4,5,6,7,8]. The anti-proliferative activities of different *Commiphora* species have also been reported [9]. However, the information regarding the compounds responsible for myrrh toxicity remains lacking. Recently, the adverse effects of high doses of the essential oils of myrrh in mice have been reported [10]. Meanwhile, a clinical study has also reported miscarriage in women who used a large amount of myrrh during pregnancy [11].

An investigation into the cytotoxicity of natural products can lead to the discovery of new anti-cancer agents. Hence, it is of the utmost importance to study the developmental toxicity of myrrh in appropriate in vitro and in vivo model systems, to investigate the cytotoxic compounds of this valuable traditional medicine. Many animal model systems are being used to assess the toxicity of newly designed compounds, natural extracts, and medicines. Recently, zebrafish have emerged as an excellent in vivo model system, not only for studying human diseases but also for the testing and target validation of a large number of compound libraries [12,13,14]. Herein, we describe the isolation of two furano-sesquiterpenoid compounds and report the cytotoxic activity against different cancer cell lines and zebrafish embryos. To the best of our knowledge, to date, there have been no reports concerning the cytotoxic activity of 2-methoxyfuranodiene and 2-acetoxyfuranodiene and this is the first cytotoxic evaluation of both compounds using in vitro cell lines and an in vivo zebrafish model.

## 2. Results

### 2.1. Identification of Compounds

**Figure 1:**
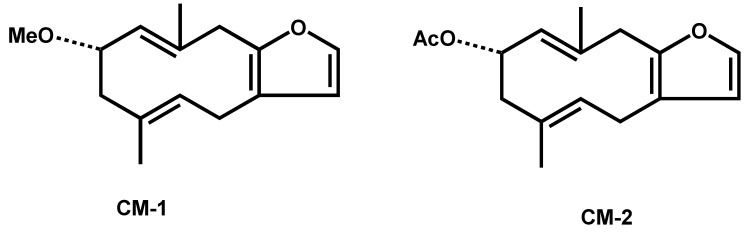
Chemical structures of 2-methoxyfuranodiene (left) and 2-acetoxyfuranodiene (right) isolated from the chloroform fraction of *C. myrrh.*

By employing 1D and 2D-NMR, mass analysis, and a comparison with literature data, the two isolated compounds were identified (Figure 1).

Compound 1: 2-methoxyfuranodiene: white crystalline: C_16_H_22_O_2_. The structure was established with the help of 1D-NMR, 2D-NMR, COSY, NOSY, EIMS, and IR, the data for which has been compared with the published data [15].

Compound 2: 2-acetoxyfuranodiene: white crystalline: C_17_H_22_O_3_. The structure was established with the help of 1D-NMR, 2D-NMR, COSY, NOSY, EIMS, and IR, the data for which has been compared with the published data [15].

### 2.2. Cytotoxic Activity

**Figure 2:**
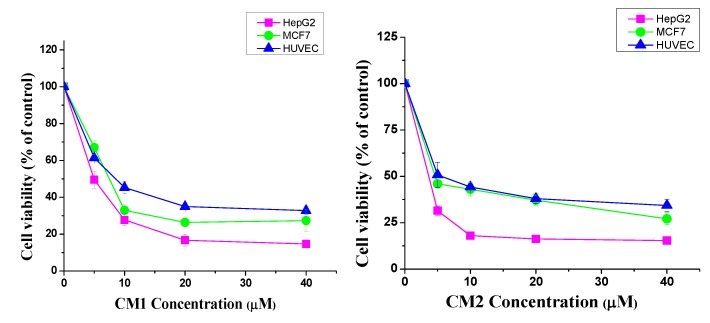
Cytotoxic effects of compounds CM1 and CM2 on different human cell lines. The viability was measured by an MTT assay. Cells were cultured as described in the methods and treated with various concentrations of CM1 and CM2 (1–40 µM) for 48 h. The Student’s t-test was used to calculate the statistical differences. The data are presented as the mean ± S.D. (* *p* < 0.05, ** *p* < 0.01, and *** *p* < 0.001 were considered significant compared to the control) for the three independent experiments carried out in triplicate.

To assess the cytotoxicity of compounds CM1 and CM2, an MTT assay was performed. As shown in Figure 2, both compounds presented high cytotoxic activity, thereby significantly decreasing the number of viable cells in a concentration-dependent manner. The dose-dependent inhibition rates of cell viability in HepG2, MCF7, and HUVEC cells are depicted in Figure 2. The median inhibitory concentration (IC_50_) values of both compounds against the tested cells are presented in Table 1. The HepG2 cell line was highly sensitive to the cytotoxic effect, therefore it was selected for further assays in this study.

### 2.3. Cell Cycle Analysis

**Figure 3:**
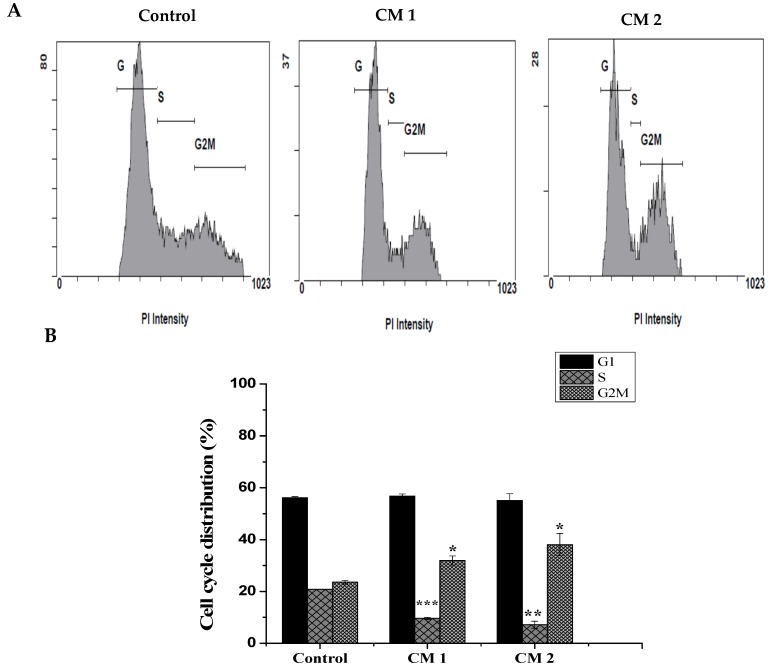
Compounds CM1 and CM2 induced cell cycle arrest. HepG2 cells were exposed to the IC_50_ for 48 h and analysis was determined as described. (**A**) Distribution of cell phases after treatment with compounds CM1 and CM2. (**B**) Quantitative analysis of data obtained. Data represented as the mean ± SD (n = 3). (* *p* < 0.05, ** *p* < 0.01, *** *p* < 0.001 vs. control)

To determine whether the decreasing number of live cells produced by the two compounds was due to cell growth inhibition, the cell cycle distributions of the control and treated cells were examined by flow cytometry after 48 h of incubation. As shown in Figure 3, the percentage of cells in the S phase significantly decreased from 20.8 ± 0.04% in the control cells to 9.6 ± 0.4% and 7.1 ± 1.3% in the HepG2 cells treated with CM1 and CM2, respectively. Consequentially, the percentage of cells in the G2/M phase increased from 23.5 ± 0.74% to 31.9 ± 1.7% and 38 ± 2%, respectively. Our results indicate that both compounds inhibit cells proliferation in the S phase and caused a significant G2/M arrest. Collectively, these results suggest that the anti-proliferative effects of these compounds are associated with arrest in the cell cycle.

### 2.4. Apoptosis/Necrosis Assessment Using Flow Cytometry

**Figure 4:**
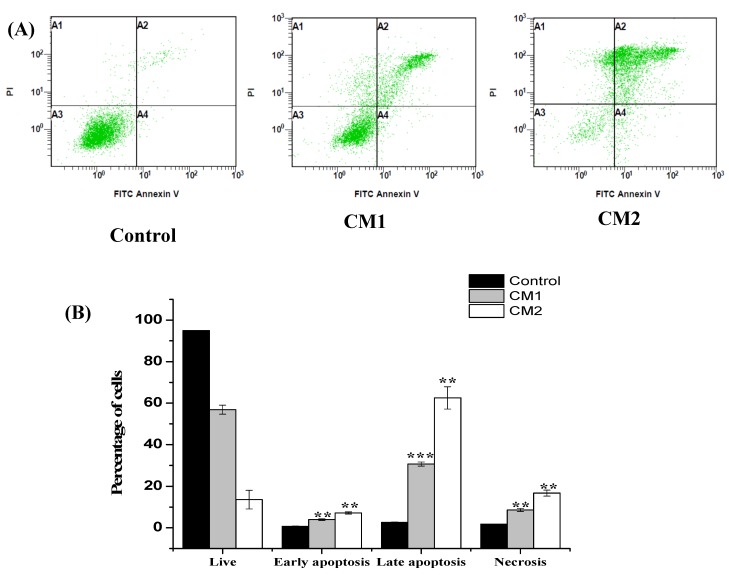
HepG2 cell death after treatment with compounds CM1 and CM2. Cells were exposed to the IC_50_ for 48 h, and cells undergoing either apoptosis or necrosis were assessed using co-staining with Annexin-V/FITC-PI dyes. (**A**) Representative scatter plots of PI (*y*-axis) vs. FITC-Annexin V (*x*-axis); live cells (A3) were negative for both dyes, (A4) early apoptotic cells were positive for only FITC -Annexin V, (A2) late apoptotic cells were stained with both dyes and(A1) Necrotic cells were stained with PI only. (**B**) Percentage of viable, early apoptotic, and late apoptotic + necrotic cells. Data are presented as the means ± SD of the triplicate experiments. ** *p* < 0.05 and *** *p* < 0.01 vs. control.

Next, we examined the mode of cell death induced by CM1 and CM2, to clarify the mechanisms behind the anti-proliferative effects of both compounds using flow cytometry with Annexin V-FITC/PI staining. HepG2 exposure (48 h) to both compounds resulted in a significant change in the early and late apoptotic cell populations. We found that the treatments led to a significant increase in the early and late apoptotic + necrotic cells, from 0.7 ± 0.14% and 2.62 ± 0.19% in the control cells to 3. 91 ± 0.42% and 30.74 ± 1% in the CM1-treated cells and 7.13 ± 0.55% and 62.56 ± 5.4% in the CM2-treated cells, respectively (Figure 4). As a result, both compounds were proven to possess strong apoptotic induction abilities against HepG2 human liver cancer cells.

### 2.5. Developmental Toxicity of 2-Methoxyfuranodiene and 2-Acetoxyfuranodiene in Zebrafish Embryos

The zebrafish embryos were treated with serial dilutions (0.1 to 30 µM) of both compounds individually. Treating the zebrafish embryos with different concentrations resulted in early and late lethality in zebrafish embryos. At concentrations higher than 50 µM, CM1 and CM2 treatments induced 100% embryonic lethality within 12 h of exposure. Generally, CM2 had a higher toxicity to zebrafish embryos compared to that of CM1. The LC_50_ values for CM2 and CM1 were 15 and 40 µM, respectively.

### 2.6. 2-Methoxyfuranodiene and 2-Acetoxyfuranodiene Inhibited the Formation of Angiogenic Blood Vessels during Zebrafish Embryonic Development

**Figure 5:**
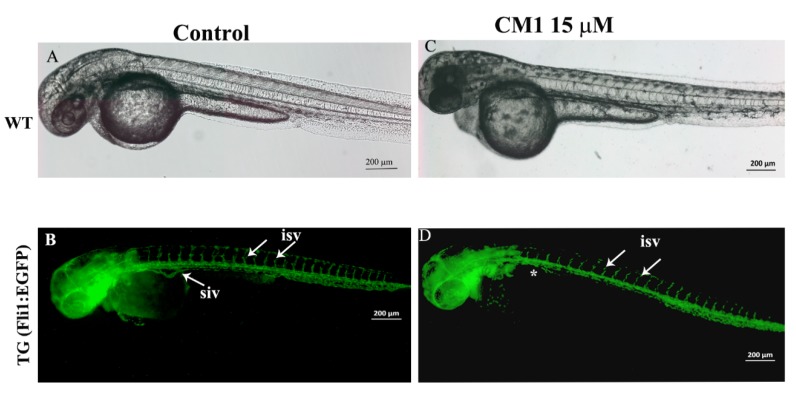
2-methoxyfuranodiene inhibited the secondary angiogenic blood vessel formation in zebrafish embryos. (**A**) Bright field image of a Tg(fli1:EGFP) embryo that was exposed to 0.1% methanol as a control. The embryos developed normally. (**B**) The fluorescent image of a Tg (fli1: EGFP) embryo at 72 hpf. The green color indicates all the blood vessels. The white arrows show the inter-segmental blood vessels in the trunk of the mock-treated zebrafish embryo, which were the first angiogenic blood vessels that formed at around 48 hpf. The emergence of the second angiogenic blood vessels can be seen on the trunk of the control embryo. (**C**) The bright field image of a Tg (fli1: EGFP) embryo treated with 10 µM of CM1. The bright field image shows the normal development of the embryos. (**D**) The fluoresce image of a Tg (fli1: EGFP) zebrafish embryo treated with 15 µM of CM1 showed the normal formation of the inter-segmental blood vessels. However, the treated embryos do not have the sub-intestinal vein blood vessels (indicated by white asterisks).

**Figure 6:**
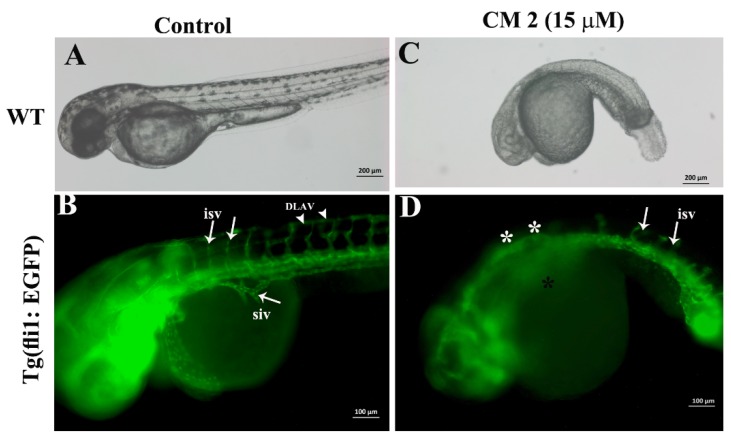
2-acetoxyfuranodiene decreased the formation of angiogenic blood vessels during zebrafish embryonic development. (**A**) Bright field image of a Tg (fli1: EGFP) embryo that was exposed to 0.1% methanol as a control. The embryos developed normally. (**B**) The fluorescent image of a Tg (fli1: EGFP) embryo at 72 hpf. The green color indicates all the blood vessels. The white arrows show the inter-segmental blood vessels in the trunk of the control embryo, which were the first angiogenic blood vessels that formed at around 48 hpf. The emergence of the second angiogenic blood vessels can be seen on the trunk of the control embryo. (**C**) A Tg (fli1: EGFP) embryo treated with 15 µM of CM2. The bright field images show that CM2 at 15 µM affected the normal development of zebrafish embryos. The embryos were much smaller in size, the body was curved, and there was no pigmentation. (**D**) The CM2 treatment disrupted the inter-somatic blood vessel formation in the treated zebrafish embryos, the majority of the inter-somatic blood vessels did not form (white asterisks), and some blood vessels formed but they did not grow and failed to connect to dorsal longitudinal anastomotic vessel. Similarly, the sub-intestinal vein did not form in the transgenic zebrafish embryos that were treated with CM2.

The Tg(fli1:EGFP) zebrafish embryos were exposed to equal concentrations of either compound to obtain the comparative anti-angiogenic activity. The anti-angiogenic activities of CM1 and CM2 are presented in Figure 5 and Figure 6, respectively. The control embryos developed normally (bright field image, Figure 5A). The inter-somatic blood vessels (white arrows in Figure 5B), which are angiogenic blood vessels, developed and grew normally in the control embryos. The zebrafish embryos that were treated with CM1 (15 µM) also developed normally up to 48 hpf (Figure 5C, bright field image). CM1 did not interfere with the inter-somatic blood vessel formation process in the treated zebrafish embryos, and so a normal development and growth of inter-somatic blood vessels was observed (white arrows in Figure 5D). However, the development of secondary angiogenic blood vessels was severely affected by CM1. The presence of a sub-intestinal vein is quite clear in the control embryos (white arrow in Figure 5C), but the sub-intestinal vein failed to develop in CM1-treated embryos (Figure 5D asterisk).

On the other hand, compound CM2 showed more efficient anti-angiogenic activity compared to that of CM1. As shown in Figure 6, CM2 affected the normal development of zebrafish embryos at a concentration of 15 µM. The embryos were much smaller in size, the body was curved, and there was no pigmentation (bright field, Figure 6C). CM2 also disrupted the inter-somatic blood vessel formation in the treated zebrafish embryos. At 48 hpf, the formation and development of the inter-somatic blood vessels in the control embryos were not affected (Figure 6B). However, the formation of inter-somatic blood vessels in zebrafish embryos that were exposed to CM2 (15 µM) were severely affected. As is evident in Figure 6D, the majority of inter-somatic blood vessels did not emerge from the dorsal aorta (Figure 6D, asterisk), some blood vessels formed but they did not grow and failed to connect to the dorsal longitudinal anastomotic vessel. The formation and growth of the sub-intestinal vein was normal in the control embryos (Figure 6B). However, the sub-intestinal vein did not form in 100% (n = 30) of the CM2-treated zebrafish embryos.

## 3. Discussion

In recent years, the global increase in the incidence of cancer has had serious impacts on human health. In this regard, new therapeutic compounds derived from natural products could be a good option for avoiding adverse effects. Historically, *C. myrrh* has been widely used in traditional medicine for the treatment of several illnesses. Its therapeutic properties have been supported by practice and evidence-based research on terpenoids (especially furano-sesquiterpenes), which are the active compounds present in myrrh essential oil [16]. Moreover, phytochemical investigations concerning *C. myrrh* resin have shown it to contain heerabolene, acadinene, elemol, eugenol, cuminaldehyde, and numerous furano-sesquiterpenes, including furanodiene, furanodienone, curzerenone, and lindestrene [17]. Substantial furano-sesquiterpenoid compounds are present in the exudates of myrrh, and approximately 20 different types have been isolated and identified [18,19,20]. In this study, two pure compounds from the chloroform fraction of 80% *v/v* ethanol extract of *C. myrrh* resin were isolated using column chromatography. These two active compounds, identified as 2-methoxyfuranodiene and 2-acetoxyfuranodiene, which are members of the furano-sesquiterpenoid family, have been previously isolated from *C. myrrh* gum [21]. However, their cytotoxic activity on human cancer cells is yet to be elucidated. Therefore, the present study aimed to investigate and explore the anti-proliferative potential of these two compounds on different cancer cells, with a focus on the different types of cell death induced. Based on the IC_50_ values, the results showed that CM2 was more active than CM1 in the tested cell lines, with HepG2 liver cancer cells displaying the most sensitivity (Table 1).

There are different modes of cell death: apoptosis, a genetically controlled or programmed process, and necrosis, a non-programmed or accidental process [22]. One feature of cancer cells is apoptosis evasion and targeting apoptosis can terminate the uncontrolled growth of cancer cells and, hence, is considered the most successful non-surgical treatment. Many known anti-cancer drugs target different apoptotic pathways [23], and several natural compounds derived from plants affect apoptotic pathways that are blocked by cancer cells via various mechanisms [24]. Flow cytometry was utilized in this study to detect apoptosis/necrosis, which can be determined by the exposure of phosphatidylserine (PS) on the surface of apoptotic cells, another hallmark of apoptosis induction [25]. The loss of control over the normal cell cycle is another hallmark of cancer cells. Additionally, the cell cycle is a mechanism that is gaining increasing interest as an anti-cancer target [26]. In our study, an evaluation of the cell cycle phase distribution using flow cytometry was employed to determine whether the compounds altered the cell cycle of HepG2 cells. The results indicated that the percentages of treated HepG2 in the S phase decreased, whereas those in the G2/M phase increased.

In fact, several studies have shown that myrrh components induce apoptosis and arrest the proliferation of cancer cells [27]. In addition, apoptosis induction and the arrest of cell cycle progression were observed to be exerted by the compounds isolated from *C. myrrh* [28].

The furano-sesquiterpene class of compounds, which are known to possess interesting biological activities, contains a wide range of chemical compounds derived from natural products [29,30]. When the anti-cancer activities of furano-sesquiterpenes and the derivatives isolated from soft coral were tested against different types of cancer cells (leukemia, prostate, lung, breast, and cervix), some of these compounds were found to demonstrate promising activity against leukemia and prostate cancer cell lines [31]. Additionally, another furano-sesquiterpene isolated from soft coral was investigated to verify its apoptotic effects. Arepalli *et al.* found that the isolated compound inhibited the proliferation of several human cancer cell lines, mediated apoptosis, and induced cell cycle arrest in human leukemia cells (THP-1) [32].

The newly designed drugs that will be used to treat various human ailments, must first be tested in suitable animal models to assess the effective dose ranges and safety of the drugs. Various animal models are currently in use to evaluate the toxicity of chemically synthesized or natural products. The zebrafish has emerged as a very powerful tool for investigating the in vivo developmental toxicity of compounds on a large scale [33,34,35]. The small size, ease of genetic manipulations, and relatively economical cost has paved the way for zebrafish to be the best organism for human disease models [36,37,38,39,40,41,42,43,44,45,46,47,48,49,50,51,52,53]. The zebrafish developmental toxicity study revealed the chronic toxicity of CM1 and CM2. Continuous exposure, for more than three days, resulted in the total lethality of exposed embryos. However, the general trend indicated that CM2 was more toxic compared to CM1 in zebrafish embryos. The in vivo toxicity correlated with the in vitro toxicity of these compounds in human cancer and normal cell lines. Studies related to the assessment of the toxicity profile of *C. myrrh* in any animal model are very limited, and there are no reports prior to this study that investigate the developmental toxicity of *C. myrrh* (or any compounds isolated from it) in zebrafish embryos. Prolonged use of *C. myrrh* has already been attributed to recurrent miscarriage and abdominal pain in one clinical case [11]. Zebrafish embryonic death, caused by exposure to furano-sesquiterpenoids isolated from *C. myrrh*, warrants that a careful dose monitoring system should be tested to avoid any adverse effects on fetus development. Various studies have reported the anti-angiogenic profile of myrrh within in vitro or in vivo systems [54,55,56,57]. During zebrafish embryonic development, the inter-somatic blood vessels and sub-intestinal vein are considered angiogenic blood vessels (which emerge from pre-existing blood vessels) and hence provide a quantitative tool to measure the angiogenic activity of natural products or synthetic compounds in live zebrafish embryos [58,59,60,61]. CM1 impeded the development of the sub-intestinal vein in the treated embryos. CM2 exhibited potent anti-angiogenic activity, as the angiogenic blood vessels, inter-segmental vessels, and sub-intestinal vein did not form in CM2-treated embryos.

In the present study, 2-methoxyfuranodiene and 2-acetoxyfuranodiene were found to be associated with anti-cancer effects, apoptosis, and cell cycle arrest in human liver cancer cells. These results validate previous studies where furano-sesquiterpene compounds exhibited obvious inhibitory effects on cancer cell proliferation. The molecular mechanisms associated with the anti-cancer activity of these compounds needs further investigation. However, the present findings suggest that the anti-proliferative activity of the compounds may be associated with their potential to induce apoptosis and inhibit angiogenesis.

## 4. Materials and Methods

### 4.1. Plant Material Collection and Compound Isolation

*C. myrrh* oleo-gum-resin was purchased from (Yasin Spices, Sana’a, Yemen) The plant identity was verified by Prof. Ramzi Mothana. A voucher specimen No. 12486 was deposited in the College of Pharmacy, Herbarium of Pharmacognosy Department, King Saud University, Riyadh, Saudi Arabia.

#### 4.1.1. Preparation of the Crude Extracts

Air-dried oleo-gum resin of C. myrrh (1.5 kg) was extracted several times using a Soxhlet apparatus with 3 L of ethanol for 7 h. The acquired crude ethanolic extract was filtered, concentrated under reduced pressure using rotatory evaporator to give 54 g, the total alcoholic extract was suspended in H_2_O and successively partitioned with n-hexane, chloroform (CHCl3), and n-butanol (BtOH) to yield 5 g of n-hexane, 15 g of chloroform, and 10 g of butanol fractions.

#### 4.1.2. Compound Isolation and Identification

The chloroform fraction (5 g) was applied onto the top of silica gel packed column (72 g, 80 × 3 cm). Elution began with 3% ethyl acetate: n-hexane, and the polarity was increased with ethyl acetate in gradient mode of elution analysis giving 18 fractions (20 mL each). Based on TLC behavior, the collected similar fractions were pooled together to yield eight main fractions. Fraction C (70 mg), eluted with 5% ethyl acetate:n-hexane, was re-chromatographed in a silica gel column (7.2 g, 60 × 1 cm) using chloroform: n-hexane solvent system. Elution mode from the column was gradient. According to their TLC behavior, similar fractions were collected to give seven sub-fractions. Sub-fraction 4, eluted with 20% CHCl3: n-hexane, yielded compounds CM-1 (25 mg) and CM-2 (28 mg). Several spectroscopic techniques were performed for structure elucidation including 1D and 2D-NMR.

### 4.2. In Vitro Biochemical Studies

#### 4.2.1. Cytotoxic Activity Test for the Isolated Compounds (MTT Assay)

Several previous studies have been shown that the MCF-7 and Hepg2 cell lines showed sensitivity to the myrrh constituents [9]. To determine the cytotoxic activity of compounds CM1 and CM2, an MTT assay was employed. Briefly, 1 × 10^5^ of HepG2 (liver) and MCF-7 (breast) cancer cells and normal HUVEC (human umbilical vein endothelial cells) cells were plated in tissue culture plates (24-well). After 24 h of incubation at 37 °C in 5% CO_2_, the cells were treated with various concentrations of both compounds (1–40 µM) for 48 h. To each well, 0.1 mL µL MTT (5 mg/mL) was incubated with cells for 2–4 h at 37 °C in 5% CO_2_. After incubation, acidified isopropanol (1 mL, 0.01 N HCL) was added to each well (forming soluble formazan), before the solution was placed on a shaker for 15 min. A microplate reader (Bio-Tek, Elx-800, Winooski, VT, USA) was used to record the optical density absorbance of the converted MTT at 570 nm. Untreated cells were considered as controls, while vinblastine was used as a positive control. For each compound tested, the IC_50_ (concentration that decreases the growth of viable cells to half) was generated from the dose-response curves. Cell Viability (%) = (O.D. of treated sample)/(O.D. of untreated sample) × 100%.

#### 4.2.2. Apoptosis Detection by Flow Cytometry (Annexin V-FITC/PI Staining)

HepG2 cells were seeded in a 6-well plate at a density of 1 × 10^6^ cells/well. After 24 h, the cells were treated with CM1 and CM2 at the IC_50_ concentration. Following exposure (48 h), both adherent and floating cells were collected by trypsinization and washed with ice-cold PBS. The pellet was re-suspended in ice cold 1× Annexin binding buffer (1 × 10^6^ cells/mL) and incubated with 5 µL of Annexin V-FITC solution (Molecular Probes, CA, USA) and 5 µL of propidium iodide (PI, 50 µg/mL). The samples were mixed gently and incubated for 15 min in the dark. Thereafter, a binding buffer (400 μL) was added to each tube, and the results analyzed by flow cytometry (Cytomics FC 500; Beckman Coulter, Brea, CA, USA). Data collection and analysis were performed using CXP software V. 3.0.

#### 4.2.3. Cell Cycle Phase Analysis

After HepG2 cells were treated with the IC_50_ for 48 h, only adherent cells were harvested, washed with ice-cold PBS, and fixed with 70% (v/v) ethanol for 4 h. Thereafter, cells were centrifuged, washed with ice-cold PBS, and incubated in 50 μL RNaseA (100 μg/mL) for 15 min. The final concentration (50 μg/mL) of propidium iodide (PI) was added and the cells were incubated in the dark at room temperature for 20 min. Cell cycle phase distributions were analyzed by a FACS Scan Flow Cytometer (Cytomics FC 500; Beckman Coulter, Brea, CA, USA). The fractions of cells in the G1, S, and G2/M phases were determined from the cell cycle distributions.

### 4.3. In Vivo Toxicity Assessment Using Zebrafish Embryos

#### 4.3.1. Animals

Wild type (AB/Tubingen tab-14) and transgenic Tg(fli1: EGFP) adult zebrafish were obtained from the Zebrafish International Resource Center (ZIRC University of Oregon, Eugene, OR, USA). The fish were maintained in the Animal Facility of the Bioproducts Research Chair, College of Science, Department of Zoology. The adult fish were fed and bred following the guidelines in ‘The Zebrafish Book’ [62].

#### 4.3.2. Toxicity Screening of Zebrafish Embryos

Embryos from wild type or transgenic zebrafish were obtained by natural pair wise breeding, as described previously [63]. Wild type zebrafish embryos were treated with serial dilutions of 0.1, 0.3, 0.9, 1, 3, 9, and 30 µM, to assess the quantitative toxicity and LC_50_ values of the compounds on developing zebrafish embryos. The transgenic Tg (fli1:EGFP) zebrafish embryos were exposed to sub LC_50_ values, to check the activity of compounds on angiogenesis (development of blood vessels).

#### 4.3.3. Angiogenesis

To assess the anti-angiogenic potential of the isolated compounds, the transgenic zebrafish line Tg (fli1: EGFP) was used. The endothelial cells (which make blood vessels) continuously express a green fluorescent protein under the fli1 promoter [64]. At six hours post-fertilization (hpf), the embryos were exposed to serial dilutions of compounds CM1 or CM2. The effects on the formation of blood vessels were checked in live zebrafish embryos, by observing the embryos using a Zeiss Axio Observer D1 Inverted fluorescent microscope paired with an FITC filter. Images were taken using ZEN software. The number of blood vessels in the control group were counted and used as a reference to quantify the number of missing blood vessels in the treated embryos.

#### 4.3.4. Microscopy and Imaging

A Zeiss Axio Observer D1 Inverted fluorescent microscope was used to capture live images. ZEN software, provided by Zeiss, was used to process the images.

### 4.4. Statistical Analysis

All experiments were conducted independently and in triplicate. The results are presented as the mean ± standard deviation (SD). Statistical comparisons and charts were constructed using origin Lab software (version 8, Massachusetts, USA). Significant differences (*p* < 0.05) were determined using a Student’s *t*-test. LC_50_ values were calculated using probit analysis, as described by Finney [65].

## 5. Conclusions

When new bioactive compounds are investigated to elucidate their potential to treat cancer, their ability to inhibit cell proliferation and induce apoptosis are two of the major characteristics examined. Our findings indicate that the two tested compounds exerted potent cytotoxic effects and induced apoptosis. In addition, the compounds possess an ability to inhibit angiogenesis, suggesting the anti-cancer potential of these compounds, which should be further examined.

## Figures and Tables

**Table 1:** Comparison of the IC_50_ values of the chloroform extract and fractionated compounds against MCF-7, HepG2, and human umbilical vein endothelial cells (HUVEC) cell lines.

Extract/Compound	Cell Lines and IC_50_ (µM)		
	HepG2	MCF-7	HUVEC
**CM1**	4.8 ± 0.3	7.5 ± 0.2	8.5 ± 0.9
**CM2**	3.6 ± 0.6	4.4 ± 0.2	4.9 ± 1.1
**Vinblastine**	1.4 ± 0.2	1.2 ± 0.3	3.1 ± 0.4
